# Canakinumab for refractory RA: a case report

**DOI:** 10.31138/mjr.29.3.170

**Published:** 2018-09-27

**Authors:** Nikolaos Marketos, Ilias Bournazos, Dimitrios Ioakimidis

**Affiliations:** 1 Department of Rheumatology, University Hospital of Linköping, Linköping, Sweden,; 2 Rheumatology Outpatient Department, Henry Dunant Hospital Centre, Athens, Greece

**Keywords:** Rheumatoid arthritis, joint erosion, Canakinumab

## Abstract

Rheumatoid arthritis is a common autoimmune disease leading often to joint destruction and reduced quality of life. We report a case of a young woman with rheumatoid arthritis with fever and rapid, destructive joint involvement verified with magnetic resonance imaging. She had failed therapy with methotrexate and leflunomide, anti-TNF, IL-6 inhibitor, B cell depletion and IL-1RA. Her laboratory results remained insignificant despite the aggressiveness of her disease. In this case, the patient only partly responded to anakinra but developed side effects, and therefore was switched to Canakinumab that led to sustained remission.

There are no clear biomarkers or other clues in order to separate early in the beginning of the disease course if a polyarticular inflammatory spectrum can be IL-1β driven. The young age of the patient at onset of disease, its aggressive course, inflammatory fever without significant laboratory inflammatory markers but with polyarthritis affecting small joints, may raise the suspicion of an IL-1β-driven disease and alert the treating rheumatologist to the use of IL-1β inhibitors early in the disease course.

## INTRODUCTION

Rheumatoid arthritis is an autoimmune disease characterized by chronic inflammation of the joints. If untreated, it leads to destruction of the joints, severe disability and premature mortality.^1^ Apart from tumor necrosis factor (TNF), another proinflammatory factor implicated in the disease mechanism is interleukin 1 (IL-1). It causes inflammation and joint erosion and inhibits the tissue repair process shown in vitro.^2^ IL-1 induces production of matrix metalloproteinases (MMPs) that lead to cartilage destruction and bone resorption and causes synovial inflammation and pannus formation by promoting direct contact between macrophages and activated T-lymphocytes. It has therefore been considered a potential target for biologic Disease-Modifying Anti-Rheumatic Drugs (bDMARDs) such as Kineret (Anakinra®) and Canakinumab (Ilaris®).^3^

## CASE DESCRIPTION

**Figure 1.**
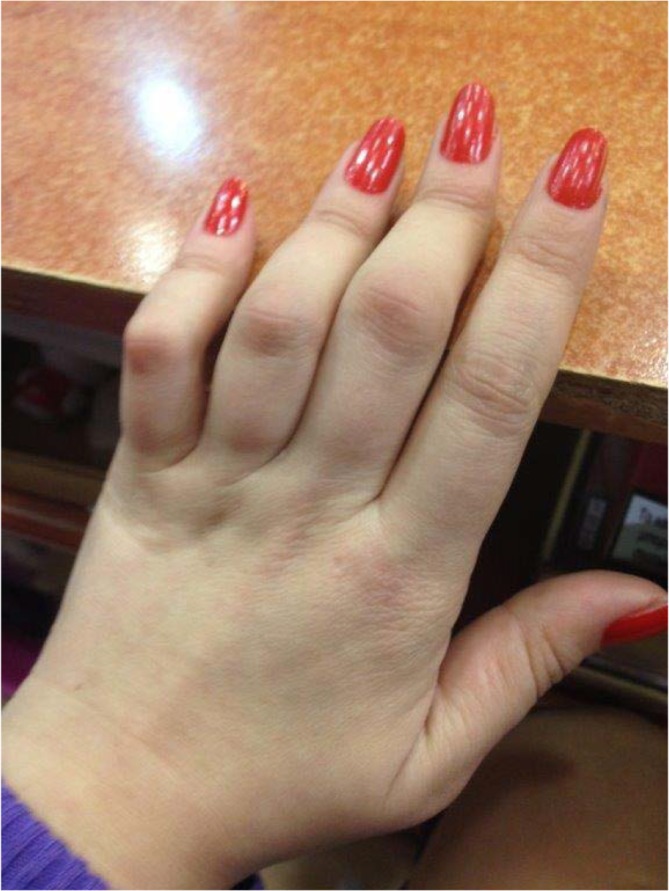
Swan-neck and boutonnière deformities as well as tenosynovitis, evidence of an aggressive disease. Picture taken before the administration of Canakinumab.

A 32-year old woman presented to the Rheumatology Outpatient Department of Henry Dunant Hospital Centre in Athens, Greece, in April 2014 suffering from polyarthritis (**Figure 1**). Her aunts from her father’s side and her grandfather suffered from rheumatoid arthritis and both her mother and sister had Hashimoto. The disease began in 2011 with fever and polyarthritis.

At the time of presentation, she had already tried and failed Methotrexate, Leflunomide, Etanercept, Golimumab, Infliximab and Tocilizumab.

**Figure 2.**
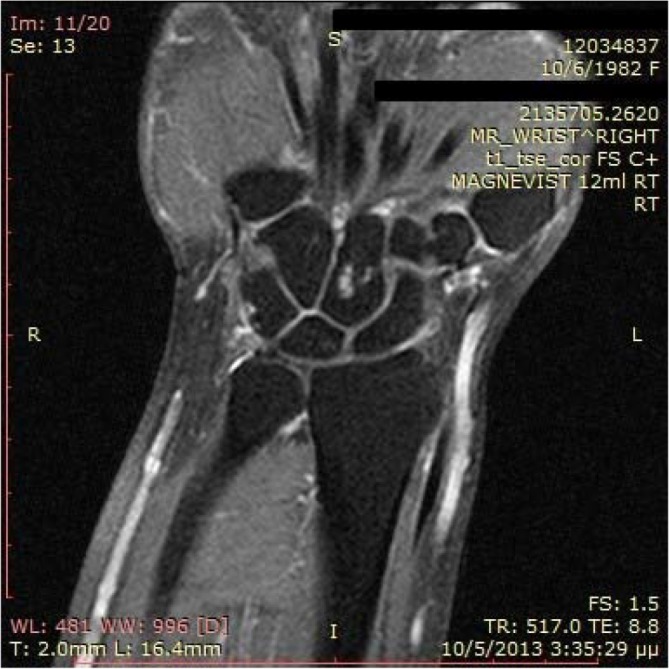
MRI with T1 sequence and use of magnetic contrast media of the patients’ right wrist indicating erosions in multiple sites.

Magnetic Resonance Imaging of the hands showed erosions, cysts and tenosynovitis (**Figure 2**). X-ray of the left wrist showed erosions of the styloid. It was then decided to change treatment to leflunomide (Arava®) 20 mg p.os in combination with Kineret (Anakinra®) 100 mg s.c.

Due to disease progression, rituximab (Mabthera®) was then tried. The first infusion was given in November 2014, followed by the second infusion with a 2-week interval. At the follow-up visit after four months, deterioration of the synovitis in all the metacarpophalangeal (MCP) joints in both hands was noted.

As the young patient’s condition was not improving despite aggressive treatment, a change in mechanism of action was then considered. The clinical approach was that this rheumatoid arthritis, resistant to all above mentioned therapy, maybe was more of auto-inflammatory origin; therefore more IL-1 driven than autoimmune. Ever since January 2016 IL-1b inhibitor Canakinumab (Ilaris®) was seriously considered. It was in April 2016 when Ilaris® was first administered. After the second subcutaneous injection, great improvement was mentioned regarding painful and swollen joints without any side-effects, situation unchanged even now.

## DISCUSSION

IL-1 consists of two similarly structured parts (a and b) and has a functional receptor on all cell types, as well as a decoy receptor on neutrophils, monocytes and B lymphocytes.^3^ Its function is regulated in four different ways: by a true endogenous receptor antagonist (IL-1Ra), soluble receptors (IL-1sRII), a decoy receptor (IL-1 RII) and natural antibodies mostly to IL-1a.^2^ Pro-inflammatory IL-1 b is transformed to IL-1b through caspase-1 in inflammasome with properties on macrophages, keratinocytes, fibroblasts, mastocytes, endothelial and neuronal cells.^4^

Synthesis and release of IL-1b depends on 2 consecutive signals. These are, in normal circumstances, started by damage-associated molecular patterns (DAMPs). These can either be pathogen associated-molecular patterns (PAMPs); for example, bacterial RNA or lipopolysaccharides, or irritants of the endogenous system, like uric acid or heat shock proteins. Synthesis of pro-IL-1b as well as components of the protein complex inflammasome NLRP3/cryopyrin is initiated by the first signal. The second signal is needed for the assembly of the inflammasome and activates caspase-1, which consecutively releases active IL-1b from pro-IL-1b.^5^

Canakinumab is a humanized monoclonal antibody against IL-1b with peak concentration of 7 days and a half-life of 22–33 days. A study of 472 patients with RA revealed a best ACR20, 50 and 70 result with the administration of 150 mg subcutaneously every 4 weeks.^4^ The authors used a predictive model of ACR response 30 months after a single administration of 2mg/kg canakinumab, namely 150mg subcutaneously. ACR 20 escalated from 60% after one month to 70% in 30 months, ACR 50 from 40 to 60% and ACR 70 from 30–40% respectively according to this simulation of probability for achieving ACR, converting the binary variables to categorical values; the results are significantly different from placebo. Its efficacy as well as safety and tolerability are established even in a 3-month phase II trial on RA patients.^6^

The drug was approved for the treatment of auto-inflammatory diseases, mainly cryopyrin-associated periodic syndromes, Adult Onset Still’s Disease (AOSD), Familial Mediterranean Fever (FMF), tumor necrosis factor receptor–associated periodic syndrome (TRAPS), and hyperimmunoglobulin D syndrome /mevalonate kinase deficiency (HIDS/MKD), although its efficacy was also studied in RA, DM2, COPD and age-related macular degeneration.^7–8^ IL-1 is even implicated in inflammatory conditions considered comorbid to rheumatoid arthritis, such as cardiovascular diseases and diabetes mellitus type 2. Therapy with Canakinumab seems to be effective even in TNF-a failures, although long-term efficacy in preservation of joint function is not known.^9^ It is therefore often used off-label in multiple indications. A recent study from Italy investigating Anakinra and Canakinumab use between 2008 and 2016 showed that the latter was used in 56% of cases off-label in 105 treatment courses. Seventy-six from a total of 475 treated patients (14.4%) reported adverse events (AEs) and 10 (1.9%) serious adverse events (SAEs), revealing a good safety profile of these medicine.^10^

In conclusion, aggressive rheumatoid arthritis in young patients demands proper and efficient treatment in order to avoid joint deformation. Despite aggressive treatment with biologics, some patients continue to suffer. Canakinumab can be a good option for those patients, and can be used outside its known indications. It would be useful to monitor these patients forward to estimate the effect of the drug on disease proliferation and radiologic progression in order to establish the best way for the patients to benefit from its use.
